# Creating space for theory when codesigning healthcare interventions

**DOI:** 10.1111/jep.13720

**Published:** 2022-06-14

**Authors:** Reema Harrison, Éidín Ní Shé, Deborah Debono, Ashfaq Chauhan, Bronwyn Newman

**Affiliations:** ^1^ Centre for Health Systems and Safety, Australian Institute of Health Innovation Macquarie University Sydney New South Wales Australia; ^2^ Graduate School of Healthcare Management RCSI University of Medicine and Health Sciences Dublin Ireland; ^3^ Centre for Health Services Research University of Technology Sydney Ultimo New South Wales Australia

**Keywords:** evaluation, health services research, healthcare, patient‐centered care

## 1 DESIGNING HEALTHCARE INTERVENTIONS AND THE ROLE OF END USERS AND STAKEHOLDERS

Improving healthcare by designing and testing theory‐based interventions is widely recognised as gold‐standard practice. Theory ‘presents a systematic way of understanding events or situations. It is a set of concepts, definitions, and propositions that explain or predict these events or situations by illustrating the relationships between variables.’^1^ Using this definition, theory provides scaffolding for intervention design by clarifying how and why particular phenomena or behaviour occur, and the factors and mechanisms that might affect change.^2^ By using theory to guide intervention design and its mechanisms of action, resulting interventions may have a stronger chance of success in creating their intended change.^3^, ^4^ The value of using and operationalising theory fully in healthcare interventions has long been recognised.^4^ Clearly articulating intervention techniques and their underlying theory facilitates their replication and evaluation.^5^ Improving interventions is also contingent upon understanding how key mechanisms contribute to outcomes and where to make changes. In contrast to the value ascribed to using theory in intervention design, the experiences and perspectives of individuals who may be likely to benefit from a given intervention have only recently been considered as an essential element for intervention design. With rapid acceleration in the use and value awarded to participatory methods in healthcare intervention design, those designing healthcare interventions must consider how to integrate knowledge from theory with that from individuals with lived experiences.^6^, ^7^, ^8^

Increasing onus on the need for codesigned healthcare interventions is notable across health systems internationally.^9^, ^10^ Codesigned interventions are those that are created by and with people who have lived experiences of the health issue, including patients, families, the public, healthcare staff and others.^7^ Involving people for whom health interventions are designed *in their design* is now considered essential in many health systems, reflected in the requirements of national healthcare and medical research funding schemes for example, in the UK and Australia.^11^, ^12^, ^13^ By codesigning interventions, health agencies seek to develop approaches that tackle issues of greatest importance to end users and that meet their needs.^12^ The result has been rapid acceleration in the number of interventions that have been codesigned across all areas of health, including those that promote health behaviour change, novel service delivery models, health policy and more.^14^, ^15^, ^16^ Rising interest in creating interventions through codesign has illuminated the lack of consideration given to deductive, theory‐based approaches to intervention development and the role that theory can play in inductive, codesigned approaches. We explore the opportunities for integrating theoretical knowledge into codesigned health interventions and the practical implications for health services and policy researchers.

## 2 WHAT IS THE ROLE OF THEORETICAL KNOWLEDGE IN CODESIGN?

Commentary on codesign in healthcare has largely been preoccupied with its process. A plethora of studies and editorial pieces have explored how to facilitate the creation of codesigned change, how to ensure equity and accessibility, and describe the resulting interventions.^17^, ^19^ It is notable that while codesigned interventions in healthcare are widely reported, few reports outline the health impacts of their interventions, their implementation and sustainment.^16^, ^20^ Discussion of the extent to which codesigned interventions harness theory and how to introduce theory in healthcare codesign processes is lacking. The benefits of integrating theory in codesign are apparent; application of theory may be used to clarify the mechanisms by which an intervention will work and the primary and secondary outcomes that require assessment to determine intervention success. In these ways, using theory can support codesigned interventions to demonstrate if they work, which is necessary for their implementation and evaluation. But for researchers seeking to utilise theory in codesign, the question remains: how can theory be introduced in codesign while privileging the voices of codesign members who are experts by experience?

## 3 CURRENT APPROACHES TO APPLYING THEORY WHEN CODESIGNING INTERVENTIONS

**Figure 1:**
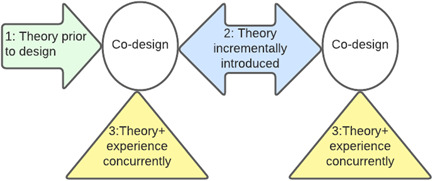
Approaches to introducing theory in codesign

Theory can be introduced at various stages of codesign processes, depicted in Figure 1. It is also possible that new theory or theory‐elaboration may result from the knowledge produced through codesign. Process research by leaders in the codesign community (within and beyond healthcare) delineates three broad approaches that enable the integration of existing theory in codesign at its outset or during the process. Each approach provides a strategy for synthesising inductive and deductive methods.

i. *Theory before design*
  Before commencing the design, or in the early stages of a design process, theoretical information about the phenomena and how to affect change can be provided to codesign members by the design and/or research team. codesign is then used to build from existing theory and to tailor interventions.^21^ This has been described as an abductive approach because codesign members reimagine existing knowledge to envision something new; the result can be considered a theory‐inspired intervention.^21^ Such an approach positions theory as a probe to evoke or elicit a response from codesign members and facilitates theory‐building through co‐design.^22^ Underpinning intervention codesign with a specified theoretical approach or model may be achieved using full‐scale generative toolkits that have been widely used to support codesign members in their efforts.^23^, ^24^ Generative toolkits support facilitated collaborative activities by including materials such as cards to sort, props for role modelling, diaries or logs, 2‐D and/or 3‐D models.^25^ Healthcare case examples demonstrate the application of these types of codesign tools to enhance interpersonal relationships and interactions, create equity and address power imbalance.^26^, ^27^ There is an opportunity for researchers to purposefully develop codesign tools that are underpinned by theory. Such an approach may provide a stronger theoretical underpinning to the resulting intervention, but in doing so raises considerations for codesign teams.
ii. *Theory introduced incrementally*
  Incremental introduction of theory throughout a codesign process is an approach that provides a space for the voices and perspectives of codesign members to come ahead of theory, and for theory to act as a scaffold for emerging ideas. Several strategies are exemplified in current literature to introduce theoretical information in this way. A strategy used by our team to bring in theory progressively at different points throughout the co‐design process was employed in codesigning patient safety interventions and is drawn from design literature that uses theory as a toolkit.^22^, ^28^ In each codesign session, ideas are initially generated by people with lived experience. The resulting ideas are used by the research team to explore relevant theory and existing works to create a mini ‘toolkit’ to inform and guide thinking amongst codesign members in the subsequent codesign session. The mini toolkits provide accessible theoretical information relevant to the emerging ideas or concepts that progressively inform the intervention design.
iii. *Theory and experience informing design concurrently*
  Progressing the strategy of introducing both theoretical knowledge and experiential knowledge throughout the design process, there are recent examples of healthcare codesign in which theory is considered concurrently to experiential knowledge. In a concurrent model, a team would synthesise theoretical knowledge from existing evidence alongside gathering new experiential information with codesign members. An example of a concurrent approach has been documented in recent work to coproduce a final set of priorities for a digital health intervention.^29^ Through several focus groups, people with lived experiences discuss their perspectives with researchers, which are subsequently explored in relation to theoretical knowledge and evidence that had been identified via a concurrent systematic review.^29^ Using these strategies, the type of theoretical knowledge introduced into the codesign or coproduction process is determined by the ideas emerging from its members.

## 4 PITFALLS WHEN INTEGRATING THEORY AND WAYS FORWARD

Integrating theory is important in codesigned healthcare interventions when the goal is to implement, evaluate, improve, and potentially scale an intervention, but presents some risks and challenges. Our experiences of bringing theory into codesign through several health service projects suggest that substantial variation in understandings of the concept and purpose of theory can be a barrier to the use of theory. It is therefore important to first gain a shared understanding of what is meant by theory and its value towards the specific project goals. Shared understanding can be achieved by using brief case studies relevant to the project goals that illustrate how theory has been applied when codesigning interventions (the how) and the benefits of doing so (the why). Cocreating basic process diagrams also enable codesign members to gain shared agreement around relationships between concepts.

One of the central challenges for codesign is ensuring that the voices of stakeholders with lived experience of a health condition or a health service are heard. Introducing theory into codesign carries the risk that theory may create power imbalances, influence the outcome of the codesign and obscure the voice of codesign members. Novel ideas and lines of thought may be restricted, and the resulting design may ultimately be generated by those with professional rather than experiential expertise. Along with many others, we have noted critical need to address power dynamics as part of a wider set of considerations for creating space and equity in codesign processes.^7^ Vigilance to the voice of codesign members and power imbalances is therefore critical when attempting to introduce theory. Techniques to alert facilitators to power imbalances can be used to direct attempts to mitigate some of this risk. A reflexive approach that focuses on relationship building and reciprocal learning between codesign members and with researchers provides the basis for enabling facilitators and codesign groups to reflect on points at which the group have made critical design decisions or deviated from the initial ideas of members.^30^ Observing video‐recordings of codesign meetings may aid facilitators and members to consider and spot power imbalances, or moments where power shifted.^31^

The use of theory in codesign and associated risks raise wider questions of the role of academic or clinician researchers who are tasked with facilitating a codesign process—what is the scope of their role, can academic/clinical subject matter experts facilitate, and what are the techniques that co‐designers can employ to mitigate these risks? In health service codesign, researchers and health service providers commonly act as facilitator but also carry professional expertise. One approach is to distinguish the role of facilitator from that of academic or clinical subject matter expert to mitigate some of the risk of power imbalance when seeking to apply theory within codesign. Techniques to achieve this include the use of academic or clinical member/s or reference group to contribute relevant theory or to adopt a coleadership model. When seeking to codesign patient safety interventions with culturally diverse communities and cancer service staff, we adopted a coleadership model in which a consumer cofacilitator partnered with an academic researcher in workshop facilitation. A central goal was to support the introduction of theory while privileging the voices of co‐design members.^30^ Our approach also highlighted the value of building capability for codesign among members as part of establishing conditions for codesign to be successful. Coleadership created a context for critical reflection and discussion between our team members that supported careful introduction of theory into the process, but also required a substantial commitment of time and to the process from all involved.

## 5 CONCLUSION

In examining the use of codesign to create healthcare interventions and the role of theory within such design, a tension between privileging the voices of codesign members and introducing theory as a facilitator may create challenges for researchers and codesign members. Process evaluation of codesign indicates that reflexive practice, coleadership models and reciprocal learning are valued strategies to support the inclusion of theory into a user‐driven approach to healthcare codesign.^30^ Strategies that require discussion, action and reflection beyond codesign meetings necessitate additional time and resource, which must be recognised and supported by leaders and researchers as an important part of preparing for and undertaking this study.^6^, ^20^

## AUTHOR CONTRIBUTIONS

Reema Harrison conceptualised the article with Éidín Ní Shé and Deborah Debono. All authors developed examples of strategies that may support integration of theory into codesign. All authors contributed sections to the draft manuscript and contributed to and agreed on the final version of the manuscript.

## Data Availability

Data sharing is not applicable to this article as no new data were created or analysed in this study.
